# The Impact of *MET* Variants in Oral Cancer Progression and Clinicopathological Characteristics

**DOI:** 10.7150/jca.106426

**Published:** 2025-02-11

**Authors:** Ping-Ju Chen, Yen-Ting Lu, Wei-En Yang, Chun-Wen Su, Lun-Ching Chang, Shun-Fa Yang, Chiao-Wen Lin, Ying-Erh Chou

**Affiliations:** 1 Department of Dentistry, Changhua Christian Hospital, Changhua, Taiwan.; 2 Department of Post-Baccalaureate Medicine, College of Medicine, National Chung Hsing University, Taichung, Taiwan.; 3 Institute of Medicine, Chung Shan Medical University, Taichung, Taiwan.; 4 School of Medicine, Chung Shan Medical University, Taichung, Taiwan.; 5 Department of Otolaryngology, Chung Shan Medical University Hospital, Taichung, Taiwan.; 6 Department of Otolaryngology, St. Martin De Porres Hospital, Chiayi, Taiwan.; 7 Department of Medical Research, Chung Shan Medical University Hospital, Taichung, Taiwan.; 8 Department of Mathematics and Statistics, Florida Atlantic University, Boca Raton, Florida, USA.; 9 Institute of Oral Sciences, Chung Shan Medical University, Taichung, Taiwan.; 10 Department of Dentistry, Chung Shan Medical University Hospital, Taichung, Taiwan.

**Keywords:** oral cancer, MET, polymorphism, susceptibility, SNP, prognosis

## Abstract

Epigenetic, genetic predisposition and epidemiological risk factors were suggested to be involved in the carcinogenesis of oral cancer. In this study, we focused on the associations of MET single-nucleotide polymorphisms (SNPs) to oral cancer susceptibility and clinicopathological characteristics. The MET SNPs rs41736, rs41739, rs1621, and rs33917957 in 1198 controls and 1318 male patients with oral cancer were analyzed with real-time polymerase chain reaction. Our results revealed that the cigarette smokers among the oral cancer patients who carried the *MET* rs1621 polymorphic variant “G” were significantly associated with lower risk to develop oral cancer [OR (95% CI) = 0.463 (0.226-0.948)]. The male oral cancer patients who with the genotypic variant “G” of *MET* rs33917957 were associated with lower risk of cell differentiated grade (p = 0.041). In the TCGA database, the MET expressions were upregulated in oral cancer tissues compared to normal tissues, and were correlated with poor cell differentiated and poorer prognoses in smoker groups. In conclusion, these novel findings underscore the role of MET genetic variants in oral cancer susceptibility, particularly in smokers, and highlight the potential of these variants for prognosis and disease prediction.

## Introduction

Oral cancer is the sixth leading cause of cancer mortality worldwide 1. About 90% of the oral cancer was originated from squamous cells, which is classified as oral squamous cell carcinoma (OSCC) 2, 3. Risk factors such as cigarette smoking, alcohol consumption, and betel nut chewing were suggested as major contributors to oral cancer 4-6. In Taiwan, the prevalence of these carcinogenic substances use in males was larger than in females, and the incidence rate of oral cancer was obviously higher in men than in women 7, 8.

MET is a proto-oncogene located on chromosome 7q31.2 which encodes the cellular-mesenchymal epithelial transition factor (c-Met) transmembrane tyrosine kinase receptor for hepatocyte growth factor (HGF) 9-11. The c-Met was suggested to play a crucial role in regulating cell proliferation, differentiation, metastasis, and apoptosis through various signaling pathways, and its aberrant expression has been implicated in many human cancers 12-14. In oral cancer, it was suggested that the MET activation may represent an early driver in oral premalignancy and may be target for chemoprevention of oral cancer, and the c-met was identified as a potential prognostic marker of cancer risk in patients with oral leukoplakia 12, 15-17. In gastric cancer, it was suggested that the MET amplification is often accompanied by human epidermal growth factor receptor 2 (HER2) overexpression, and co-expression of MET and HER2 can synergistically enhance tumor invasion, and metastasis, which is a crucial factor for poor prognosis 18.

Previous studies have associated the overexpression of MET with cancer progression and prognosis including oral cancer 15, 17, 19, and the *MET* polymorphisms were suggested to be associated with cancer development and prognosis in various cancers such as small cell lung cancer 20, gastric cancer 21, 22, hepatocellular carcinoma (HCC) 23, and papillary thyroid carcinoma 23. However, the associations and influences of *MET* polymorphisms to oral cancer tumor progression and clinicopathologic characteristics remained unclear. In the current study, we focused on four SNPs of *MET* rs41736, rs41739, rs1621, and rs33917957, and try to elucidate the associations of *MET* SNPs to oral cancer susceptibility and clinicopathologic characteristics with environmental risk factors.

## Materials and Methods

### Study subjects

A total of 1318 male patients with oral cancer and 1198 cancer-free controls were enrolled in our study. These participants who enrolled in our study were recruited during 2016 to 2021 at Chung Shan Medical University Hospital in Taichung, Taiwan. For the TNM staging of the oral cancer patients who enrolled in our study, the TNM staging were clinically staged at the time of diagnosis according to the American Joint Committee on Cancer (AJCC) 24. The rating of tumor differentiation was examined by pathologist according to the AJCC classification. This project was approved by the institutional review board of Chung Shan Medical University Hospital (IRB number: CS1-21151).

### Sample preparation and DNA extraction

For genomic DNA extraction, we collected the peripheral blood specimens from oral cancer patients and normal controls who participated in our study 25. All the samples of peripheral whole blood were preserved with EDTA containing tubes. Each sample of whole blood were centrifuged under the settings of 3000 rpm, 10 minutes. The buffy coats were collected from centrifuged whole blood specimens and further used for DNA extraction 26. Following the manufacturer's manual of QIAamp DNA blood mini kits, the genomic DNA extraction assay was performed to acquire the DNA. The Tris-EDTA (TE) buffer was applied to complete the final step of DNA elution. The extracted DNA was used as the DNA template in real-time polymerase chain reactions (PCRs).

### *MET* SNPs genotyping

Assessment of allelic discrimination for the *MET* rs41736, rs41739, rs1621, and rs33917957 SNP was performed with an ABI StepOne Software v2.3 Real-Time PCR System. The TaqMan assay was adopted for the analysis of genotyping. The final data of genotyping was analyzed and calculated with the SDS 7000 series software (Applied Biosystems, Foster City, CA, USA).

### Statistical analysis

The student's t test or Chi-squared test was performed between the patients with oral cancer and the controls to compare the age (years), betel quid chewing, cigarette smoking, alcohol drinking, tumor stage, tumor T status, lymph node status, metastasis, and cell differentiation. A p < 0.05 was suggested to present statistically significant. To compare the associations of the odds ratio (OR) with their 95% confidence intervals (CIs), and the clinical pathological statuses between the oral cancer risk and genotypic frequencies, the data was analyzed and assessed with multiple logistic regression models. All of the data in our study was analyzed and evaluated with SAS statistical software (Version 9.1, 2005; SAS Institute, Cary, NC).

## Results

The distribution of demographical characteristics in 1198 controls and 1318 male patients with oral cancer was listed in Table 1. In the current study, we observed that the distributions of age (years) < 55 was 565 (47.2%) in controls and 619 (47.0%) in oral cancer patients, and the age ≧ 55 in controls and oral cancer patients was 633 (52.8%) and 699 (53.0%), respectively. The distributions of environmental risk factors exposure between the controls and oral cancer patients were 199 (16.6%) and 985 (74.7%) in betel quid chewing (p < 0.001), 636 (53.1%) and 1110 (84.2%) in cigarette smoking (p < 0.001), and 237 (19.8%) and 626 (47.5%) in alcohol drinking (p < 0.001), respectively.

The genotype distributions of *MET* gene polymorphisms in 1198 controls and 1318 male patients with oral cancer were listed in Table 2. The highest distribution frequencies in oral cancer patients of *MET* genetic polymorphisms rs41736, rs41739, rs1621, and rs33917957 were polymorphic variant C, polymorphic variant A, polymorphic variant A, and polymorphic variant A, respectively. The logistic regression models were applied to estimate the odds ratios (ORs) with their 95% confidence intervals (CIs). Our findings revealed no significant association between *MET* SNPs and the incidence of oral cancer (Table 2). Additionally, we applied propensity score matching (PSM), a statistical technique, to evaluate the intrinsic impact of *MET* variants on oral cancer progression. After matching for age, betel quid chewing, cigarette smoking, and alcohol consumption, no significant associations were observed between *MET* variants and oral cancer incidence (Table 3).

We further analyzed the ORs and 95% CIs of oral cancer patients associated with *MET* genotyping and allele frequency among cigarette smokers. A significant association was found in those individuals who carried the *MET* rs1621 polymorphic GG genotype, with a lower risk of oral cancer susceptibility [The odds ratio (OR) (95% CI):0.463 (0.226-0.948); p = 0.035] (Table 4). Moreover, after we analyzed the odds ratio (OR) and 95% CI of clinical statuses associated with genotypic frequencies of *MET* rs33917957 in male oral cancer patients, we found that in 1110 cigarette smokers among the total 1318 male oral cancer patients, carriers who with the rs33917957 polymorphic “G” variant have a lower risk to develop poorer cell differentiated grade (p = 0.041) (Table 5).

**Figure 1:**
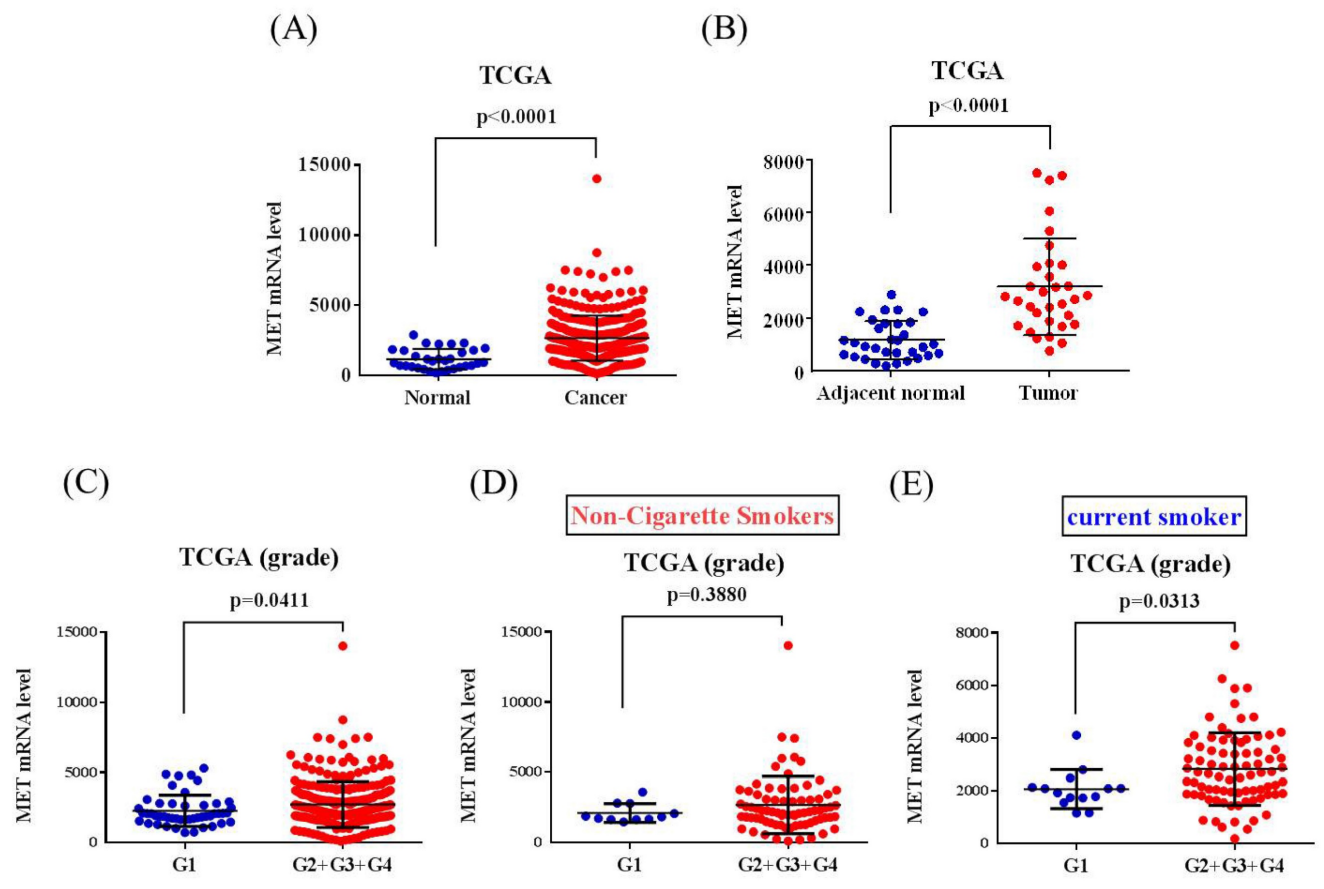
** MET levels in the head and neck squamous cell carcinoma (HNSCC) patients from TCGA database.** (A) MET levels were compared between the HNSCC tumor tissues and normal tissue. (B) MET levels were compared between the HNSCC tumor tissues and adjacent noncancerous normal tissue. (C-E) MET levels were compared between grade I (G1, well-differentiated), grade II (G2, moderately differentiated), grade III (G3, poorly differentiated), and grade IV (G4, undifferentiated or anaplastic) in (C) all patients, (D) non-cigarette smokers and (E) smokers.

**Figure 2:**
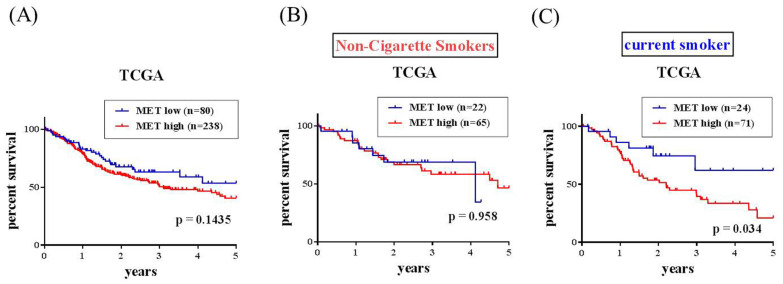
** MET expression and overall survival in the head and neck squamous cell carcinoma (HNSCC) patients from TCGA database.** MET expression and overall survival in (A) All HNSCC, (B) non-cigarette smokers (C) cigarette smoker population.

The correlations of MET expression levels with clinical significance and survival rates in head and neck squamous cell carcinoma (HNSCC) patients were further analyzed from the TCGA dataset. We observed that MET expression was prone to be upregulated in HNSCC carcinoma tissue compared with normal tissues (Figure 1A-1B). Furthermore, patients with moderately differentiated (G2) and poorly differentiated (G3) showed significantly higher MET expression in tumors compared to patients at well-differentiated (G1) both in all the HNSCC patients (p=0.0411) and the smoker group (p=0.0313) (Figures 1C-E). Most importantly, HNSCC patients who had MET^high^ tumors had shorter disease-specific survival times compared with those who had MET^low^ tumors in the smoker group (p=0.034) (Figures 2A-2C). Taken together, the above clinical data indicated that upregulation of MET is a critical event in promoting HNSCC progression.

## Discussion

In this study, we demonstrated the associations between the *MET* SNPs and oral cancer. Alcohol consumption, betel quid chewing, and cigarette smoking are the three major and well-known risk factors for head and neck cancer 27-30. In Taiwan, most of the oral cancer patients were male who with the habits of cigarette smoking and/or betel nut chewing, and over 90% of oral cancer was OSCC 31-33. Consistent with these results, in our current study, statistically significant associations of these risk factors including betel quid chewing, cigarette smoking, and alcohol drinking were found between the 1198 controls and 1318 male patients with oral cancer, respectively (p < 0.001, table 1). We further analyzed the associations of genotyping and allele frequency of *MET* SNP in oral cancer patients and normal controls.

However, no significant association was found between the oral cancer patients and normal controls, suggesting a limited effect of these *MET* SNPs to oral cancer carcinogenesis. Intriguingly, after we analyzed the genotyping and allele frequency of *MET* SNP in oral cancer among cigarette smokers, a significant association was found in *MET* rs1621 polymorphisms between the oral cancer patients and controls, suggesting a lower risk to develop oral cancer carcinogenesis of these individuals who carried the *MET* rs1621 “GG” genotype.

A previous study has suggested that the SNP rs1621 in the seed-matching sequence of *MET* was related to affect the activity of miR-199a, which mediates the downregulation of the *MET* gene through targeting the 3'-UTR 34. In a study of HCC, the variant GG genotype of *MET* rs1621 was suggested to be associated with a decreased risk for HCC, and the *MET* rs1621 polymorphism may influence susceptibility to HCC, alone and combined with miR-199a rs74723057 23. Consistent with this result, our data exhibited the same result in oral cancer among cigarette smokers. Of note, in a study of micropapillary-predominant subtype pulmonary adenocarcinoma (MPPAC), it was suggested that the c-MET protein overexpression was significantly associated with smoking status, lymphatic and venous invasion, and tumor-node-metastasis stage, but c-MET gene amplification showed no relation with any of these characteristics 35. Moreover, cigarette smoking was suggested to induce overexpression of c-Met receptor in microvessels of oral lichen planus 36, and the influence of cigarette smoking to induce overexpression of HGF in type II pneumocytes and lung cancer cells was also observed 37. These studies have indicated that the smoking status was highly linked to c-MET protein overexpression, overexpression of c-Met receptor, and overexpression of HGF in various cancers. Taken together, although the expression of c-MET protein, c-Met receptor, and HGF was not detected in our study, it can be proposed that these oral cancer patients involved in our study who smokes may have higher level of these key regulators of MET pathway. Even under the circumstances, the oral cancer patients who carried the *MET* rs1621 “GG” genotype still represented to be associated with decreased risk of oral cancer. The *MET* rs1621 to regulate the activity of miR-199a which mediates the downregulation of the *MET* gene through targeting the 3'-UTR might provide a possible mechanism to explain this phenomenon 23.

For *MET* rs33917957, although the information for rs33917957 is limited, a previous study focused on gastric cancer has suggested that the MET N375S variant genotypes (NS/SS) were associated with a significantly decreased risk of gastric cancer 22. The MET N375S (rs33917957 A>G) was revealed as a germline missense variant in exon 2 of the *MET* gene which corresponds to the semaphorin domain of the MET protein 22, and the MET N375 forms two potential hydrogen bonds, whereas S375 modeling structure retains only one hydrogen bond, thereby has weaker ligand binding affinity compared with the N375 22, 38. Another study which focused on the effect of c-Met expression on survival in head and neck squamous cell has suggested the possible role of c-Met as an early marker of poor prognosis and a hallmark of aggressive biological behavior in oral cancer 39. Compared with these results, our data showed consistency with the rs33917957 expressed in gastric cancer that the cigarette smokers of male oral cancer patients who carried the *MET* rs33917957 “AG + GG” polymorphic variants were associated with lower risk to develop moderate or poorer cell differentiated grade, and the ligand binding affinity from MET N375 to S375 may play an essential role in oral cancer. Besides, it was suggested that high expression of c-Met was associated with the primary location of head and neck carcinomas 40. The squamous cell carcinoma expressed c-Met was found to be more frequently than undifferentiated carcinoma, and positive c-Met expression was suggested to correlated with high probability of lymph node metastasis 40. Therefore, although the oral cancer patients who carried the *MET* rs33917957 polymorphisms showed no statistically significant association of clinical variables such as the clinical stage and TNM staging in our study, and most of the oral cancer patients enrolled in our study are individuals without lymph node metastasis, the *MET* rs33917957 may hence be interpreted as an early marker to evaluate cancer progression and prognosis oral cancer. However, the exact expressions of MET/HGF pathway correspond to *MET* polymorphisms and oral cancer progression and prognosis require future well-designed study to elucidate it.

In conclusion, our study first demonstrated the associations of *MET* polymorphisms to oral cancer disease susceptibility and clinical statuses. The study's findings have practical applications in both early detection and personalized treatment for oral cancer. By identifying individuals at risk based on their *MET* SNPs and considering factors such as smoking status, clinicians could offer more individualized approaches to prevention, screening, and treatment. Moreover, further research on MET expression and its role in cancer prognosis could lead to the development of new therapeutic targets.

## Figures and Tables

**Table 1:** The distributions of demographical characteristics in 1198 controls and 1318 patients with oral cancer.

Variable	Controls (N=1198)	Patients (N=1318)	p value
Age (yrs)			p=0.921
<55	565 (47.2%)	619 (47.0%)	
≥55	633 (52.8%)	699 (53.0%)	
Betel quid chewing			p < 0.001*
No	999 (83.4%)	333 (25.3%)	
Yes	199 (16.6%)	985 (74.7%)	
Cigarette smoking			p < 0.001*
No	562 (46.9%)	208 (15.8%)	
Yes	636 (53.1%)	1110 (84.2%)	
Alcohol drinking			p< 0.001*
No	961 (80.2%)	692 (52.5%)	
Yes	237 (19.8%)	626 (47.5%)	
Stage			
I+II		618 (46.9%)	
III+IV		700 (53.1%)	
Tumor T status			
T1+T2		662 (50.2%)	
T3+T4		656 (49.8%)	
Lymph node status			
N0		866 (65.7%)	
N1+N2+N3		452 (34.3%)	
Metastasis			
M0		1308 (99.2%)	
M1		10 (0.8%)	
Cell differentiation			
Well differentiated		183 (13.9%)	
Moderately or poorly differentiated		1135 (86.1%)	

Mann-Whitney U test or Chi-square test was used between healthy controls and patients with oral cancer. * p value < 0.05 as statistically significant.

**Table 2:** Genotyping and allele frequency of *MET* single nucleotide polymorphism (SNP) in oral cancer and normal controls.

Variable	Controls (N=1198) n (%)	Patients (N=1318) n (%)	OR (95% CI)
**rs41736**			
CC	371 (31.0%)	406 (30.8%)	1.000 (reference)
CT	584 (48.8%)	658 (49.9%)	1.030 (0.860-1.232)
TT	243 (20.2%)	254 (19.3%)	0.955 (0.762-1.196)
CT+TT	827 (69.0%)	912 (69.2%)	1.008 (0.851-1.194)
**rs41739**			
AA	369 (30.8%)	405 (30.7%)	1.000 (reference)
AG	584 (48.8%)	657 (49.9%)	1.025 (0.856-1.227)
GG	245 (20.4%)	256 (19.4%)	0.952 (0.760-1.192)
AG+GG	829 (69.2%)	913 (69.3%)	1.003 (0.847-1.189)
**rs1621**			
AA	902 (75.3%)	996 (75.6%)	1.000 (reference)
AG	273 (22.8%)	305 (23.1%)	1.012 (0.840-1.219)
GG	23 (1.9%)	17 (1.3%)	0.669 (0.355-1.261)
AG+GG	296 (24.7%)	322 (24.4%)	0.985 (0.821-1.181)
**rs33917957**			
AA	1036 (86.5%)	1139 (86.4%)	1.000 (reference)
AG	158 (13.2%)	171 (13.0%)	0.984 (0.781-1.242)
GG	4 (0.3%)	8 (0.6%)	1.819 (0.546-6.058)
AG+GG	162 (13.5%)	179 (13.6%)	1.005 (0.800-1.263)

The ORs with analyzed by their 95% CIs were estimated by logistic regression models.

**Table 3:** Genotyping and allele frequency of *MET* single nucleotide polymorphism (SNP) in oral cancer and normal controls after propensity score matching^a^.

Variable	Controls (N=530) n (%)	Patients (N=530) n (%)	OR (95% CI)^b^
**rs41736**			
CC	173 (32.6%)	174 (32.8%)	1.000 (reference)
CT	251 (47.4%)	265 (50.0%)	1.050 (0.800-1.378)
TT	106 (20.0%)	91 (17.2%)	0.854 (0.601-1.212)
CT+TT	357 (67.4%)	356 (67.2%)	0.991 (0.767-1.281)
**rs41739**			
AA	172 (32.5%)	174 (32.8%)	1.000 (reference)
AG	251 (47.4%)	264 (49.8%)	1.040 (0.792-1.365)
GG	107 (20.1%)	92 (17.4%)	0.850 (0.599-1.205)
AG+GG	358 (67.5%)	356 (67.2%)	0.983 (0.760-1.271)
**rs1621**			
AA	402 (75.8%)	385 (72.6%)	1.000 (reference)
AG	116 (21.9%)	137 (25.8%)	1.233 (0.928-1.638)
GG	12 (2.3%)	8 (1.6%)	0.696 (0.281-1.721)
AG+GG	128 (24.2%)	145 (27.4%)	1.183 (0.898-1.558)
**rs33917957**			
AA	457 (86.2%)	455 (85.8%)	1.000 (reference)
AG	72 (13.6%)	75 (14.2%)	1.046 (0.738-1.482)
GG	1 (0.2%)	0 (0.0%)	---
AG+GG	73 (13.8%)	75 (14.2%)	1.032 (0.729-1.461)

^a^ Propensity score matching for age, betel quid chewing, cigarette smoking, and alcohol consumption, ^b^ The ORs with analyzed by their 95% CIs were estimated by logistic regression models.

**Table 4:** Genotyping and allele frequency of *MET* single nucleotide polymorphism (SNP) in oral cancer among cigarette smokers.

Variable	Controls (N=636) n (%)	Patients (N=1110) n (%)	OR (95% CI)
**rs41736**			
CC	208 (32.7%)	336 (30.3%)	1.000 (reference)
CT	298 (46.9%)	558 (50.3%)	1.159 (0.928-1.449)
TT	130 (20.4%)	216 (19.4%)	1.029 (0.779-1.358)
CT+TT	428 (67.3%)	774 (69.7%)	1.119 (0.908-1.380)
**rs41739**			
AA	207 (32.5%)	335 (30.2%)	1.000 (reference)
AG	298 (46.9%)	557 (50.2%)	1.155 (0.924-1.444)
GG	131 (20.6%)	218 (19.6%)	1.028 (0.779-1.357)
AG+GG	429 (67.5%)	775 (69.8%)	1.116 (0.905-1.377)
**rs1621**			
AA	476 (74.8%)	846 (76.2%)	1.000 (reference)
AG	143 (22.5%)	250 (22.5%)	0.984 (0.778-1.243)
GG	17 (2.7%)	14 (1.3%)	**0.463 (0.226-0.948)^a^**
AG+GG	160 (25.2%)	264 (23.8%)	0.928 (0.740-1.164)
**rs33917957**			
AA	552 (86.8%)	962 (86.7%)	1.000 (reference)
AG	81 (12.7%)	140 (12.6%)	0.992 (0.740-1.329)
GG	3 (0.5%)	8 (0.7%)	1.530 (0.404-5.790)
AG+GG	84 (13.2%)	148 (13.3%)	1.011 (0.758-1.348)

The ORs with analyzed by their 95% CIs were estimated by logistic regression models. ^a^p = 0.035.

**Table 5:** Odds ratio (OR) and 95% confidence intervals (CI) of clinical statuses associated with genotypic frequencies of *MET* rs33917957in male oral cancer patients.

	Total (N=1318)			Cigarette Smokers (N=1110)			Non-Cigarette Smokers (N=208)		
Variable	AA (N=1139)	AG+GG (N=179)	p value	AA (N=962)	AG+GG (N=148)	p value	AA (N=177)	AG+GG (N=31)	p value
**Clinical Stage**									
Stage I+II	527 (46.3%)	91 (50.8%)	0.255	446 (46.4%)	75 (50.7%)	0.327	81 (45.8%)	16 (51.6%)	0.548
StageIII+IV	612 (53.7%)	88 (49.2%)		516 (53.6%)	73 (49.3%)		96 (54.2%)	15 (48.4%)	
**Tumor size**									
≦ T2	576 (50.6%)	86 (48.0%)	0.530	502 (52.2%)	72 (48.6%)	0.423	74 (41.8%)	14 (45.2%)	0.728
> T2	563 (49.4%)	93 (52.0%)		460 (47.8%)	76 (51.4%)		103 (58.2%)	17 (54.8%)	
**Lymph node metastasis**									
No	742 (65.1%)	124 (69.3%)	0.280	627 (65.2%)	104 (70.3%)	0.225	115 (65.0%)	20 (64.5%)	0.961
Yes	397 (34.9%)	55 (30.7%)		335 (34.8%)	44 (29.7%)		62 (35.0%)	11 (35.5%)	
**Metastasis**									
M0	1129 (99.1%)	179 (100.0%)	-	953 (99.1%)	148 (100.0%)	-	176 (99.4%)	31 (100.0%)	-
M1	10 (0.9%)	0 (0.6%)		9 (0.9%)	0 (0.0%)		1 (0.6%)	0 (2.2%)	
**Cell differentiated grade**									
Well	151 (13.3%)	32 (17.9%)	0.098	133 (13.8%)	30 (20.3%)	**0.041**	18 (10.2%)	2 (6.4%)	0.521
Moderate or poor	988 (86.7%)	147 (82.1%)		829 (86.2%)	118 (79.7%)		159 (89.8%)	29 (93.6%)	
